# A prognostic risk model for programmed cell death and revealing TRIB3 as a promising apoptosis suppressor in renal cell carcinoma

**DOI:** 10.18632/aging.205237

**Published:** 2023-11-22

**Authors:** Qingfeng Fu, Bocun Yi, Qiang Su, Yue Huang, Lin Wang, Zhihong Zhang

**Affiliations:** 1 Department of Urology, Tianjin Institute of Urology, The Second Hospital of Tianjin Medical University, Tianjin 300211, China; 2 Department of Urology, The Affiliated Yantai Yuhuangding Hospital of Qingdao University, Yantai 264000, Shandong, China

**Keywords:** programmed cell death, renal cancer, prognosis, immunotherapy response, TRIB3

## Abstract

Programmed cell death (PCD), a common modality of cell death, affects tumor development and acts as a target for tumor therapeutics. Many modalities of PCD regulate genesis, progression and metastasis of cancers, thus affecting the patients’ prognosis, but the comprehensive molecular mechanisms of PCD in tumors are lacking, especially in renal cancer. Here, seventeen PRPCDGs were identified from 1257 genes associated with thirteen PCD modalities, which were highly differentially expressed and significantly affected patients’ prognosis. Then, LASSO regression analysis of these PRPCDGs screened the 9-gene PRPCDGs risk signature in TCGA-KIRC database. The PRPCDGs risk signature was closely associated with the patients’ prognosis and presented stable prediction efficacy for 5- and 7-year overall survival (OS) in three different cohorts of renal cancer. Immune cell infiltration, immune checkpoint expression and pathway enrichment (including GO, KEGG pathway, tumor-associated pathways and metabolism-associated pathways) were significantly different in the high- or low-PRPCDGs-risk group. Finally, we illustrated that TRIB3 might be a protumor factor responsible for the elevated proliferation and invasion capacities of renal cell carcinoma (RCC) cells. In summary, the PRPCDGs risk signature was developed and showed stable prediction efficacy for the prognosis of patients and that (such as TRIB3) could be a potential target for RCC management.

## INTRODUCTION

RCC is a significant global health problem, causing a substantial number of deaths each year [1]. RCC is the most prevalent among different types of kidney cancer and constitutes at least 90% of all cases [2]. Kidney renal clear cell carcinoma (KIRC), the most prevalent type of RCC, accounts for approximately 80% of all RCC cases [3]. Unfortunately, RCC is associated with poor outcomes, and its incidence has been steadily increasing [4]. Approximately 30% of RCC patients eventually develop metastases [4]. KIRC is characterized as a highly immuno-invasive tumor, indicating its ability to evade immune surveillance [5], and the progression of renal cancer has been associated with disruptions in the tumor immune microenvironment and tumor metabolism [6]. Numerous therapeutics such as surgery, targeted therapy and immunotherapy, have been applied for the management of KIRC patients in the clinic, but their efficacy remains limited [7]. This underscores the persistent challenge in effectively treating KIRC and reveals the need to recover new therapeutic targets.

PCD is a common modality of cell death in multicellular organisms that is genetically controlled and accompanied by physiological dysfunction [8]. PCD affects various physiological and pathological processes in organisms [9]. Apoptosis, one of the earliest discovered PCD modalities, is regulated by a series of intracellular and extracellular signaling networks [9]. Both aberrant or absent apoptotic signaling and excessive expression of apoptosis inhibitors can lead to the survival of tumor cells [10]. In addition, DNA damage is a common trigger of apoptosis. Severe DNA damage and defective repair mechanisms can activate the apoptotic signaling pathway and mediate apoptosis [11]. Autophagy is another common form of PCD and is responsible for removing damaged or excess cellular components and maintaining intracellular environmental homeostasis, which plays a significant role in tumor development [12]. Autophagy was reported to be a survival pathway and quality-control mechanism to suppress tumor progression during early tumorigenesis, while when tumors progress to the advanced stage, autophagy promotes the survival, growth and metastasis of established tumors [13]. As the investigation of PCD deepens, various PCD modalities (such as pyroptosis, ferroptosis, necroptosis, cuproptosis, etc.,) are being discovered and studied. These different types of PCD have distinct characteristics and regulatory mechanisms and play vital roles in tumor development [14–17]. Thus, exploration of the potential functions, biological mechanisms and clinical relevance of various types of PCD in KIRC is needed to provide a reference for the subsequent targeted therapies.

In this study, 1257 protein coding genes associated with thirteen PCD modalities (apoptosis, autophagy, pyroptosis, ferroptosis, necroptosis, alkaliptosis, oxciptosis, parthanatos, anoikis, cuproptosis, entotic cell death, netotic cell death, and lysosome-dependent cell death) were obtained from previous studies [18] and GSEA database. Seventeen genes that were highly differentially expressed and significantly affected patients’ prognosis were screened as prognosis-related PCD genes (PRPCDGs). Subsequently, the 9-gene PRPCDGs risk signature, namely ATP6V0A4, ATP6V1C2, DCN, MT1G, MYH14, NTRK2, PROM2, TRIB3 and UCHL1, was identified by LASSO regression analysis on the basis of PRPCDGs’ expression in The Cancer Genome Atlas Kidney Renal Clear Cell Carcinoma (TCGA-KIRC). Patients’ prognosis, immune checkpoint expression, immune cell infiltration, Gene Ontology (GO) and pathway enrichment analyses (including KEGG pathway, tumor-associated pathway and metabolism-associated pathway) in the high-PRPCDGs-risk group significantly differed from those in the low-PRPCDGs-risk group. Analyses of univariate and multivariate Cox (uni- and multi-Cox) regression were then adopted and the PRPCDGs risk signature was identified as the independent risk factor. The PRPCDGs risk signature was then mirrored in two cohorts, the Cancer Genome Atlas Kidney Renal Papillary Cell Carcinoma (TCGA-KIRP) and the E-MTAB-1980, indicating that the signature has high efficacy in the prognostic prediction of RCC. In addition, in both the KIRC and KIRP cohorts, patients with progressive disease (PD) accumulated higher PRPCDGs risk scores and the nomogram model constructed by the PRPCDGs risk signature and several clinical parameters possessed a stable prediction efficacy for 5-/7-year OS. All data revealed that PRPCDGs risk signature was capable of predicting the prognosis and immunotherapy response of the patients. Finally, to further confirm the impact of PCD in RCC, we comprehensively explored the effect of TRIB3 (a hub gene in PRPCDGs) on renal tumors. TRIB3 was highly expressed in A498 cells and had a negative correlation with patients’ prognosis. Knockdown of TRIB3 decreased the proliferation potential, invasion capacity and colony formation ability of A498 cells. Meanwhile, the downregulation of TRIB3 promoted the apoptosis and autophagy process and decreased the expression levels of proteins related to DNA damage repair. In summary, our study uncovered the effect of PRPCDGs on KIRC and developed a feasible risk signature for the prediction of patients’ prognosis and therapeutic response that could be a potential target for tumor therapies.

## RESULTS

### Identification of PRPCDGs

**Figure 1:**
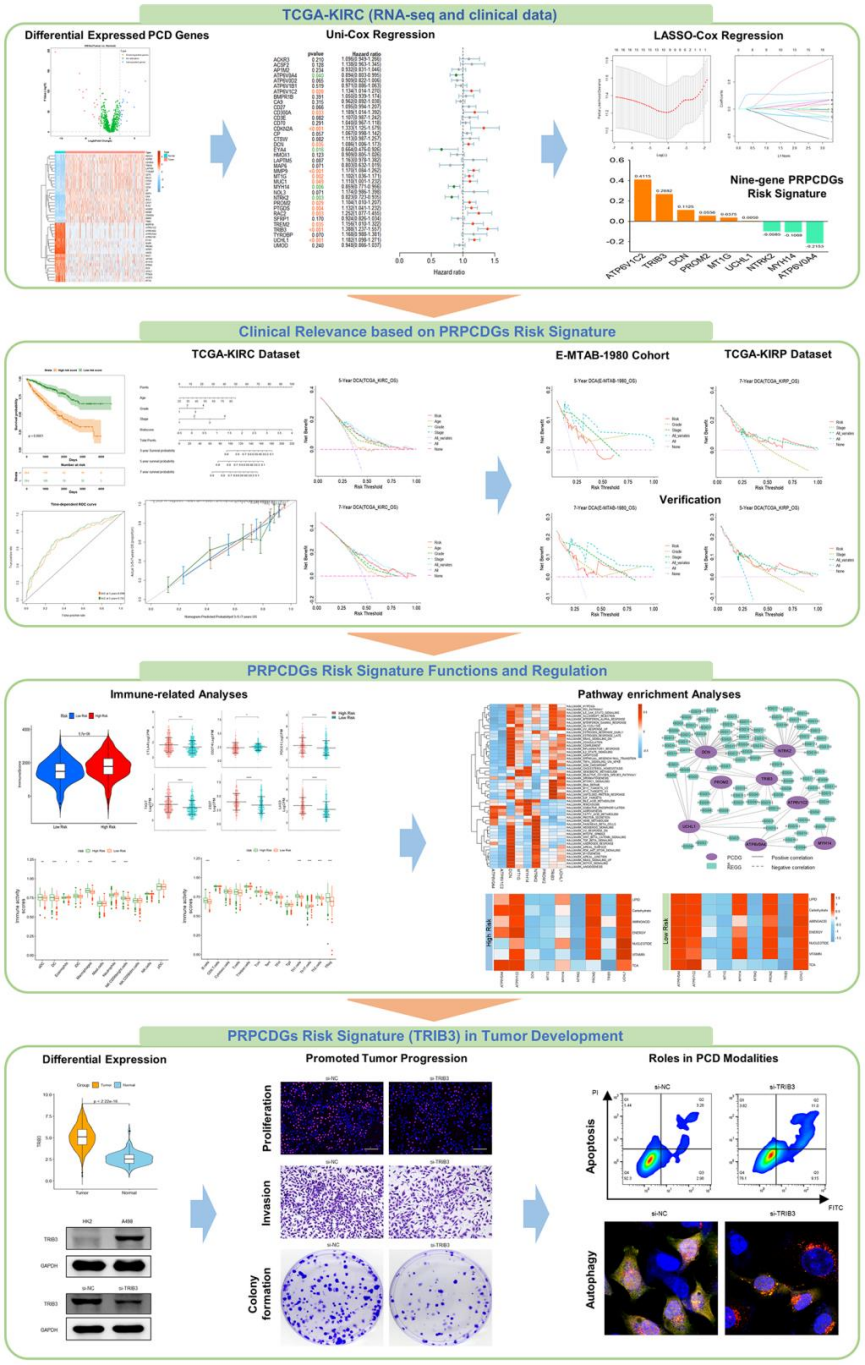
The workflow of the study.

**Figure 2:**
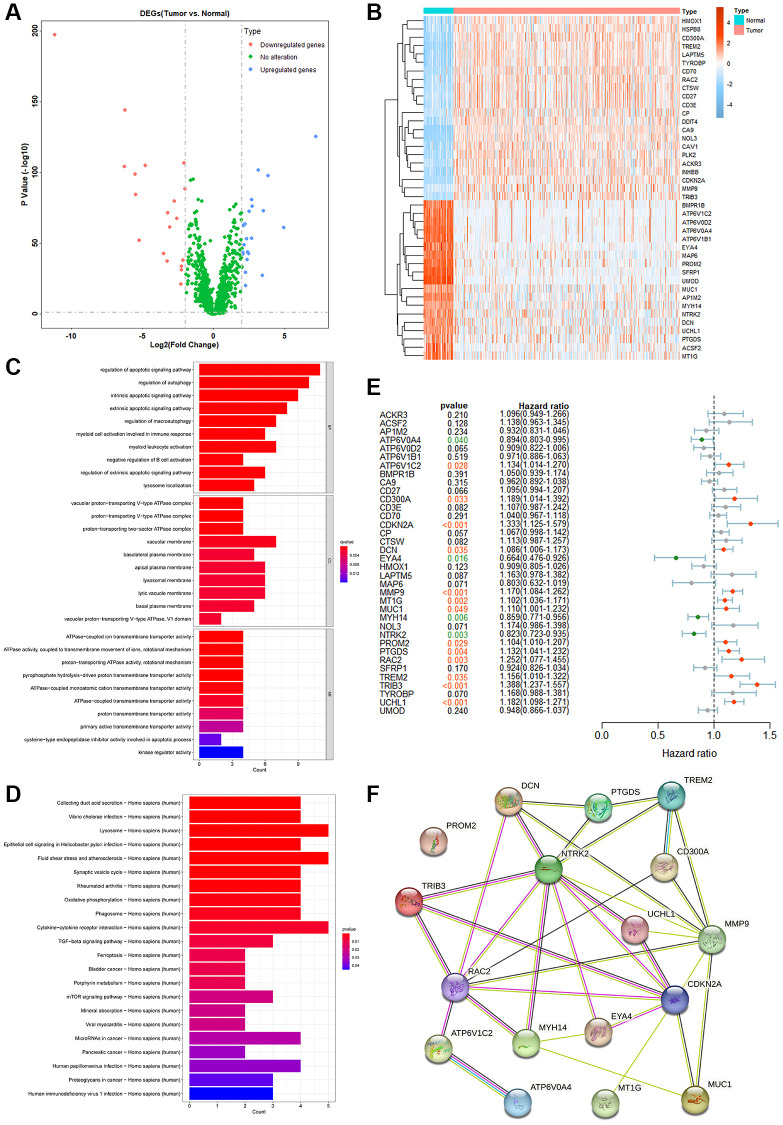
**Identification of PRPCDGs.** (**A**) The diagram shows PCD-related DEGs (|logFC| >2.0, adjusted *P*-value < 0.05). (**B**) The heatmap in the presence of mRNA expression of these PCD-related DEGs in TCGA-KIRC database. (**C**, **D**) The GO and KEGG enrichment analyses of these PCD-related DEGs. (**E**) The forest plots of prognosis-related DEGs identified by the analysis of uni-Cox regression. (**F**) The PPI of these prognosis-related DEGs (named as PRPCDGs).

The workflow of this study is shown in Figure 1. First, we obtained 1257 protein coding genes related to PCD from previous studies [18] and the GSEA database, including apoptosis, autophagy, pyroptosis, ferroptosis, necroptosis, alkaliptosis, oxciptosis, parthanatos, anoikis, cuproptosis, entotic cell death, netotic cell death, and lysosome-dependent cell death (shown in Supplementary Table 1). Forty-one differentially expressed protein-coding genes (DEGs) were screened to significantly distinguish tumor tissues from normal tissues, covering 22 significantly upregulated genes in tumor tissues and 19 downregulated genes (Figure 2A, 2B and Supplementary Figure 1A). These genes were primarily enriched in the apoptosis, autophagy and lysosome pathways in renal tumor tissues. (Figure 2C, 2D). A total of 7.71% (31 in 402) of samples had somatic mutations in these genes (Supplementary Figure 1B). A change in copy number variation (CNV) was also observed in these genes (Supplementary Figure 1C). The surv_cutpoint R-function was adopted to determine the ideal cutoff of 41 DEGs expression and the Kaplan-Meier survival curve was further depicted. The results showed that 87.8% (36 in 41) of these genes had a significant correlation with the prognosis of KIRC patients; specifically, high expression of 11 genes (AP1M2, ATP6V0A4, ATP6V0D2, CA9, EYA4, HMOX1, MAP6, MYH14, NTRK2, SFRP1 and UMOD) contributed to favorable prognosis of KIRC patients, while the high expression of the other 25 genes (ACKR3, ACSF2, ATP6V1B1, ATP6V1C2, BMPR1B, CD27, CD300A, CD3E, CD70, CDKN2A, CP, CTSW, DCN, LAPTM5, MMP9, MT1G, MUC1, NOL3, PROM2, PTGDS, RAC2, TREM2, TRIB3, TYROBP and UCHL1) led to poor outcomes in patients (Supplementary Figure 2A). Furthermore, uni-Cox regression analysis among these 36 genes was conducted and the results demonstrated that 17 DEGs had significant correlations with OS (*P* < 0.05) of KIRC patients (Figure 2E), including 4 protective factors (HR <1) and 13 risk factors (HR >1), which were termed PRPCDGs. Meanwhile, multi-Cox regression analysis (adjusted by age, grade and stage) was performed and the results showed that TRIB3, ATP6V1C2, UCHL1, NTRK2 and MYH14 were the independent factors in KIRC (Supplementary Figure 2B). The PPI network of 17 PRPCDGs is presented in Figure 2F.

**Figure 3:**
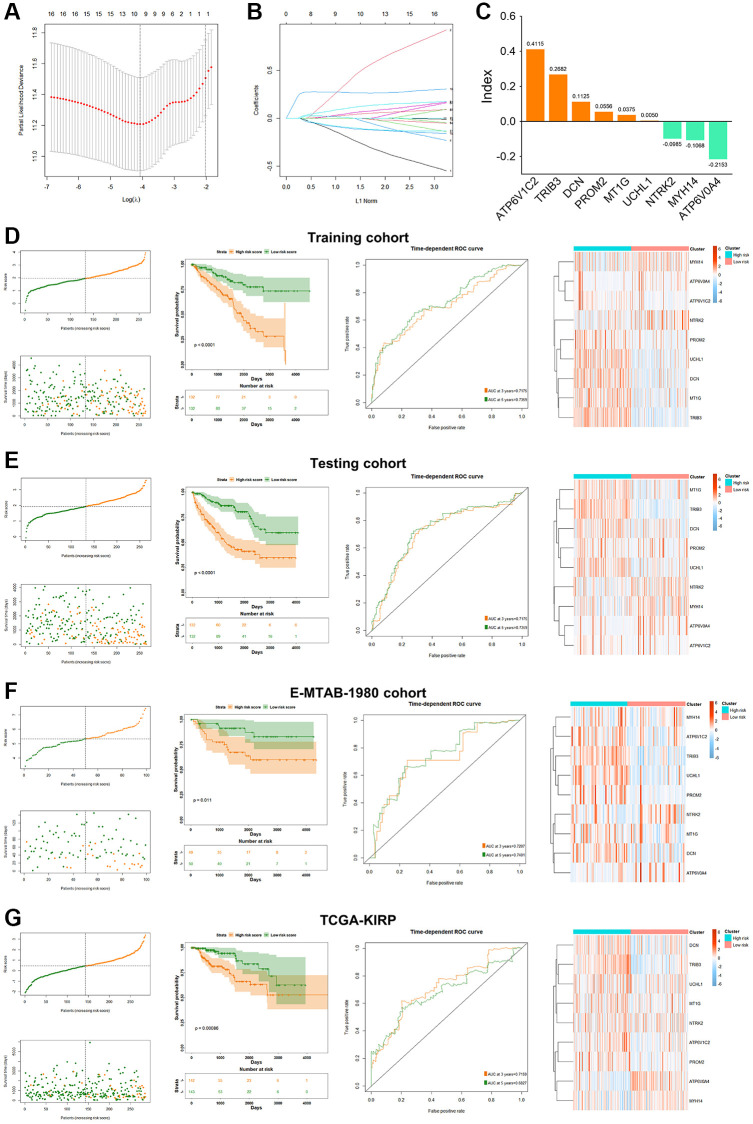
**The identification and prognostic analyses of the PRPCDGs risk signature.** (**A**, **B**) LASSO regression analyses of PRPCDGs in TCGA-KIRC training dataset. (**C**) Bar plot on LASSO regression coefficients of the PRPCDGs. (**D**) The display of risk score, OS and OS status; Kaplan–Meier curves of the high- or low-PRPCDGs-risk group; prognostic performance of PRPCDGs risk signature presented in time-dependent ROC curves; heatmap of PRPCDGs expression in these two risk groups in KIRC training dataset. (**E**–**G**) The same analyses in KIRC-testing cohort and the external validation datasets (E-MTAB-1980 and TCGA-KIRP).

To further verify the roles of PCDGs in KIRC patients, a ratio of 1:1 was adopted to divide the TCGA-KIRC dataset into training and testing groups. A risk model was established via LASSO Cox regression analysis based on 17 PRPCDGs’ mRNA expression in KIRC-training dataset. Genes with high similarity but low weights were eliminated and 9 genes with low similarity were identified in this model (Figure 3A, 3B), namely ATP6V0A4, ATP6V1C2, DCN, MT1G, MYH14, NTRK2, PROM2, TRIB3 and UCHL1. Based on the expression data and the coefficients of 9 genes, a prognostic model was constructed. The genes and their coefficients incorporated into this model are shown in Figure 3C, and the formula was as follows: PRPCDGs risk score = ATP6V1C2 × 0.41151193 − ATP6V0A4 × 0.21532181 + DCN × 0.11250123 + MT1G × 0.03749527 − MYH14 × 0.10682736 − NTRK2 × 0.09850798 + PROM2 × 0.05556780 + TRIB3 × 0.26818505 + UCHL1 × 0.00498139.

### The prognostic prediction of the PRPCDGs risk signature

For the assessment of PRPCDGs risk signature's ability to predict outcomes for patients, the survival status, Kaplan-Meier survival curves, ROC curves as well as the heatmap of PRPCDGs’ expression were assessed. In the KIRC-training cohort (Figure 3D), the median value of risk scores divided all the patients into high- and low-PRPCDGs-risk groups and rising risk scores were accompanied by an increase in mortality. The Kaplan-Meier analysis suggested that patients with low-risk scores gained more favorable outcomes than those with high PRPCDGs risk scores. The ROC curves of 3- and 5-year survival in these two groups represented a discriminative accuracy (AUC in 3-year survival = 0.7175, AUC in 5-year survival = 0.7359). The heatmap of PRPCDGs expression data in different groups revealed that the expression of ATP6V1C2, MT1G, TRIB3, DCN, PROM2 and UCHL1 was positively related to the risk scores while the other 3 PRPCDGs presented negative correlations. Furthermore, the subgroups of various clinical features (age, sex, clinical stage and pathological grade) were selected and the prognosis of patients in these two groups was calculated in various subgroups, illustrating that patients from the high-PRPCDGs-risk group confronted more unfavorable outcomes than those in the other group among most subgroups (Supplementary Figure 3), which mirrored that the PRPCDGs risk signature had a robust predictive efficacy in patients’ prognosis. Moreover, the efficacy of this predictive model was further tested in KIRC-testing and E-MTAB-1980 datasets (Figure 3E, 3F). These two cohorts presented similar results that the mortality was positively related to risk scores and patients in high-PRPCDGs-risk group confronted more unfavorable outcomes than those in the other group. ROC curves for 3- and 5-year survival showed that the AUCs were 0.7175 and 0.7359 in KIRC-testing cohort whereas 0.7207 and 0.7401 in E-MTAB-1980 dataset, respectively. In comparison to the low-PRPCDGs-risk group, the expression of ATP6V1C2, DCN, MT1G, PROM2, TRIB3, and UCHL1 was higher in high-PRPCDGs-risk group. All these findings were consistent with the KIRC-training dataset, suggesting that elevated coexpression of ATP6V1C2, DCN, MT1G, PROM2, TRIB3 and UCHL1 led to unfavorable prognosis in KIRC patients. In addition, the PRPCDGs risk signature was adopted in TCGA-KIRP cohort and showed a reliable prediction performance (Figure 3G).

### Clinical relevance on the basis of PRPCDGs risk signature

**Figure 4:**
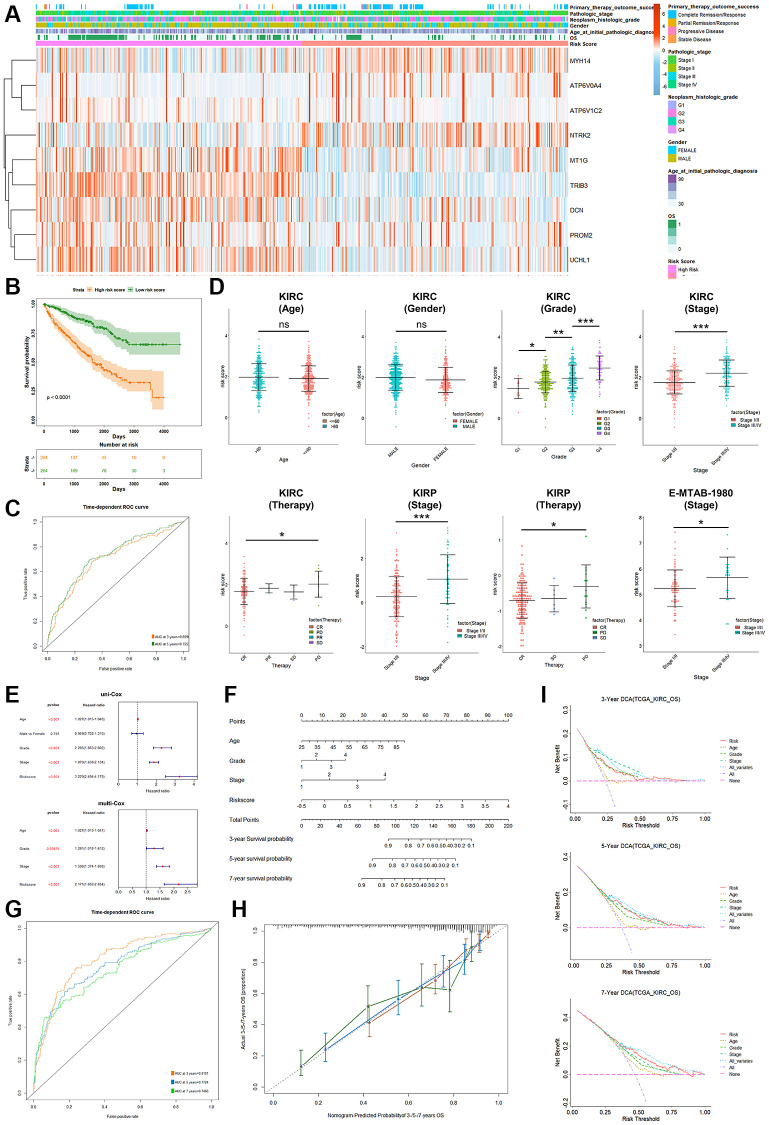
**Analysis of clinical relevance and clinical model construction based on PRPCDGs risk signature.** (**A**) Heatmap of PRPCDGs’ expression in TCGA-KIRC dataset. (**B**, **C**) Kaplan–Meier survival and ROC curves based on PRPCDGs risk signature. (**D**) Comparison of the risk score in high- and low-PRPCDGs-risk groups on gender, age, stage, grade, and therapy response in KIRC cohort and two external validation datasets (E-MTAB-1980 and TCGA-KIRP). (**E**) Uni- and multi-Cox regression analyses based on OS in KIRC cohorts. (**F**) Nomogram model of the 3-/5-/7-year survival probability of patients in KIRC cohort. (**G**) ROC curve in the presence of model’s predictive accuracy in KIRC dataset. (**H**) The calibration curve in the presence of 3-, 5-, and 7-year OS probability of the model in KIRC dataset. (**I**) DCA curve in the presence of 3-, 5-, and 7-year OS probability according to clinical models in KIRC dataset.

**Figure 5:**
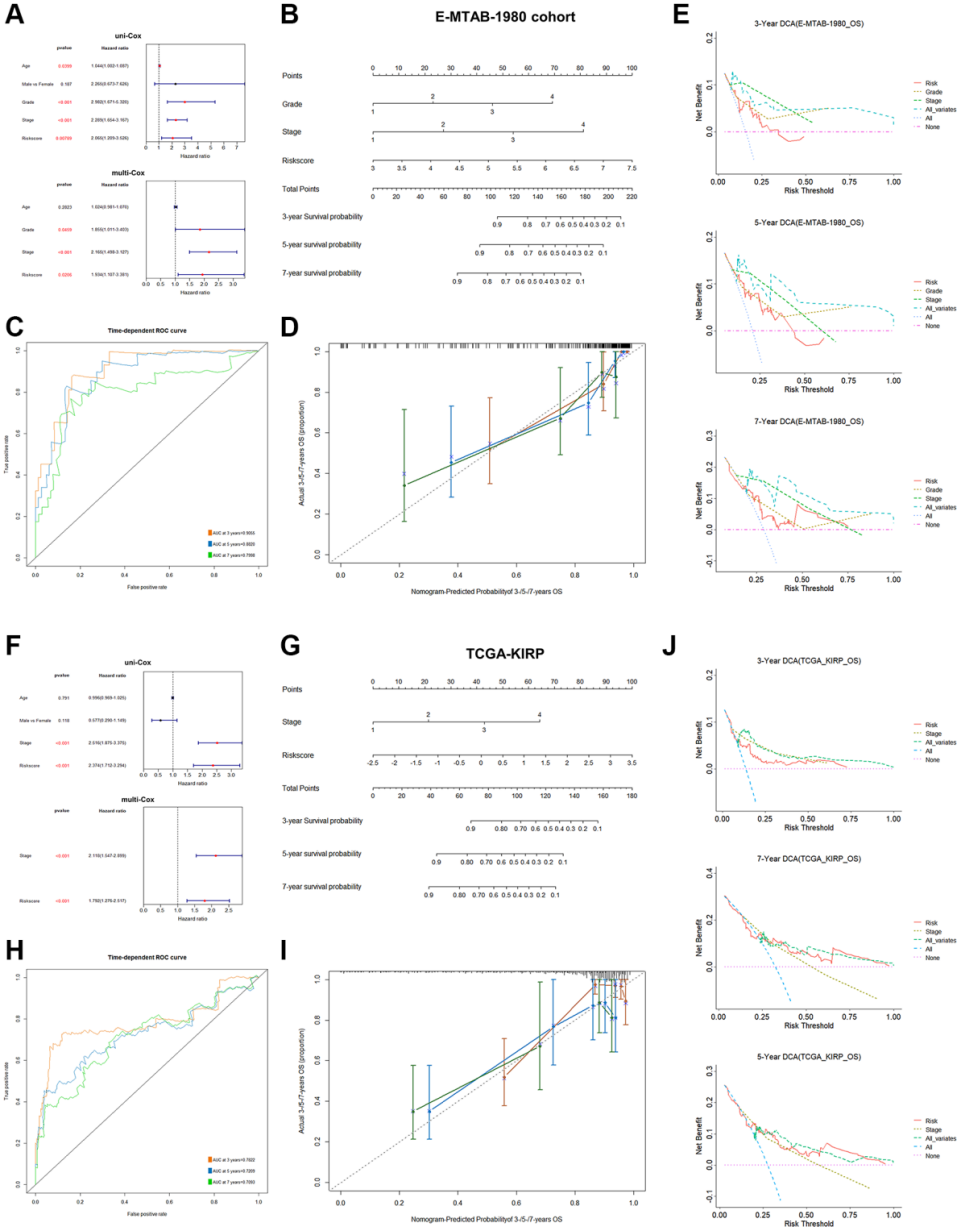
**Clinical model construction based on PRPCDGs risk signature in E-MTAB-1980 and TCGA-KIRP datasets.** (**A**–**E**) Uni- and multi-Cox regression analyses based on the OS; a nomogram model on patients’ 3-/5-/7-year survival probability; ROC curves presenting model’s predictive accuracy; calibration curves and DCA curves over 3-/5-/7-year OS probability in E-MTAB-1980 dataset. (**F**–**J**) The same analyses in KIRP dataset.

The clinical characteristics of KIRC patients were further analyzed based on these 9 PRPCDGs. In KIRC cohort, the differential expression and prognosis prediction performance of 9 PRPCDGs, and the survival difference corresponded with the results described previously (Figure 4A–4C). The correlations between risk scores and various patients’ clinical features were estimated (Figure 4D). The risk scores of KIRC patients did not differ significantly by age or gender, but they did have positive correlations with pathological grade and clinical stage. In addition, higher PRPCDGs risk scores were observed in patients with PD in the KIRC cohort, which suggested that PRPCDGs risk signature was capable of predicting the therapy response. Moreover, patients with the advanced clinical stage (Stage III/IV) in KIRP and E-MTAB-1980 datasets showed higher risk scores, and patients with PD in E-MTAB-1980 cohort represented high risk scores. Patients in KIRP and E-MTAB-1980 datasets with advanced clinical stages (Stage III/IV) displayed higher risk scores, and patients with PD from E-MTAB-1980 dataset reflected high risk scores. Uni- and multi-Cox regression analyses were carried out based on age, gender, grade, stage, and risk scores to further investigate independent predictive efficacy of PRPCDGs risk signature in KIRC cohort, revealing that the risk score was the independent prognostic factor for KIRC patients (Figure 4E). For the prediction of the likelihood of 3-/5-/7-year survival, a nomogram model was established with analysis of multi-Cox regression (Figure 4F). ROC and calibration curve analyses assessed the model’s accuracy (Figure 4G, 4H), and the DCA curves showed the highest clinical value for 5-/7-year prediction of patients’ prognosis in the model consisting by risk scores, age, grade and clinical stage (Figure 4I). A similar analysis for the PRPCDGs risk signature was conducted in E-MTAB-1980 cohort, indicating that the model (consisting of risk score, pathological grade and clinical stage) for 5-/7-year prognosis of patients possessed the best prediction value, further highlighting the ideal predictive efficacy of the model for patients’ prognosis (Figure 5A–5E). Similarly, the model consisting by risk score and clinical stage showed satisfactory prediction value for patients’ prognosis in TCGA-KIRP cohort (Figure 5F–5J), which indicated that the PRPCDGs signature could be universal in other RCC tumors.

### Immune-related analyses of patients in high- and low-PRPCDGs-risk groups

**Figure 6:**
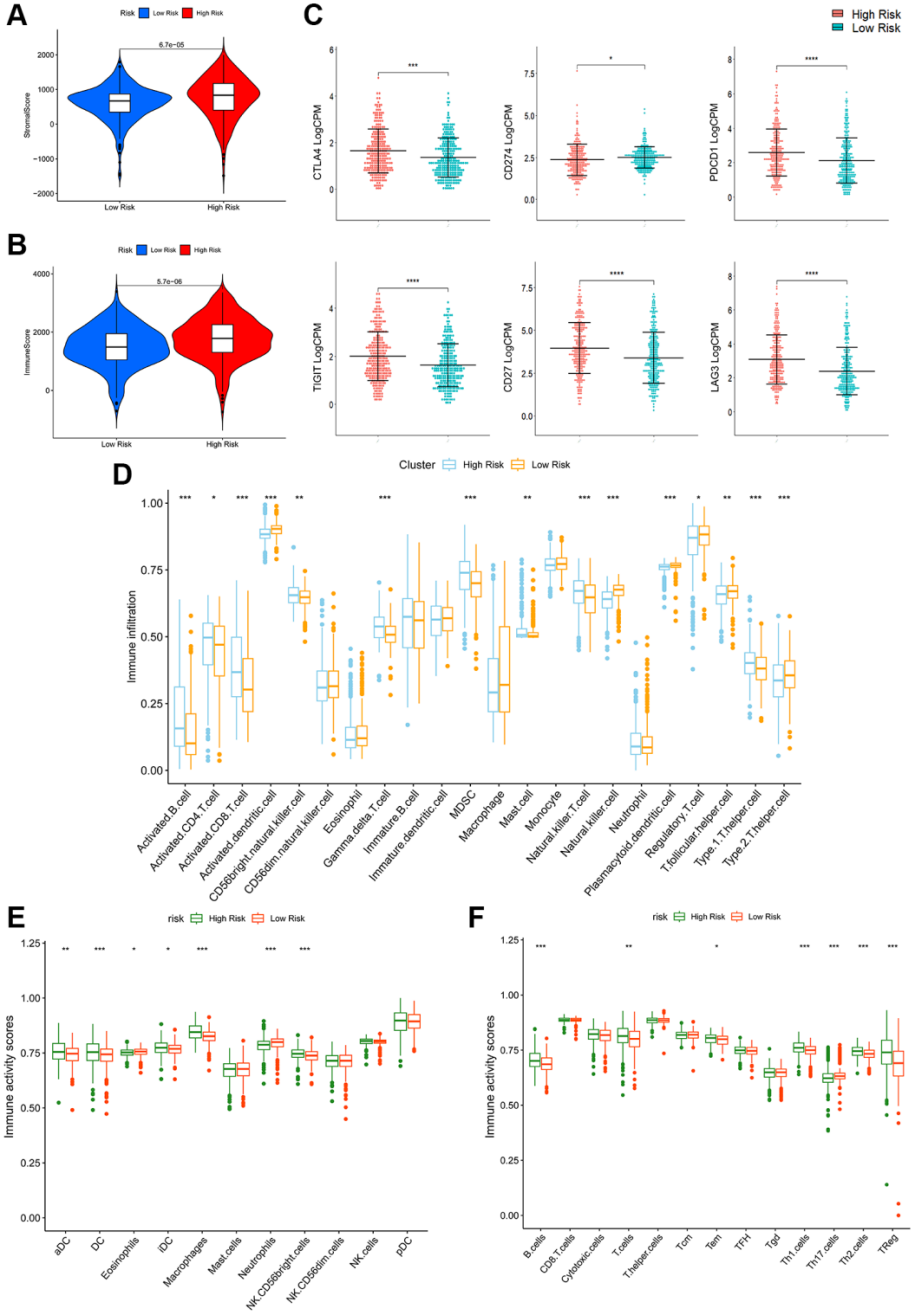
**Immune-related analyses in high- and low-PRPCDGs-risk groups in TCGA-KIRC cohort.** The stromal scores (**A**), immune scores (**B**), immune checkpoints’ expression (**C**), immune infiltration (**D**), immune activity scores of innate (**E**) and adaptive (**F**) immune cells in these two groups.

As calculated by the ESTIMATE algorithm, high-PRPCDGs-risk group had higher stromal and immune scores than the other group (Figure 6A, 6B). Additionally, assessments of immune checkpoint expression were made in these groups, revealing that high-PRPCDGs-risk group presented higher expression levels of CTLA4, PDCD1, TIGIT, CD27, and LAG3 while CD274 was less expressed (Figure 6C), suggesting that patients with a different risk level tended to show contrary responses to immunotherapy. In addition, the infiltration and activity of various immune cells were assessed. The content of most immune cells showed a significant discrepancy in these two groups (Figure 6D). As shown in Figure 6E, 6F, aDC, DC, iDC, macrophages, CD56-bright NK cells, B cells, T cells, Tem, Th1 cells, Th2 cells, and Treg cells were highly activated in the high-PRPCDGs-risk group, whereas eosinophils, neutrophils, and Th17 cells showed negative relations with risk scores in patients from TCGA-KIRC cohort.

### Potential molecular mechanisms of the PRPCDGs risk signature in TCGA-KIRC patients

**Figure 7:**
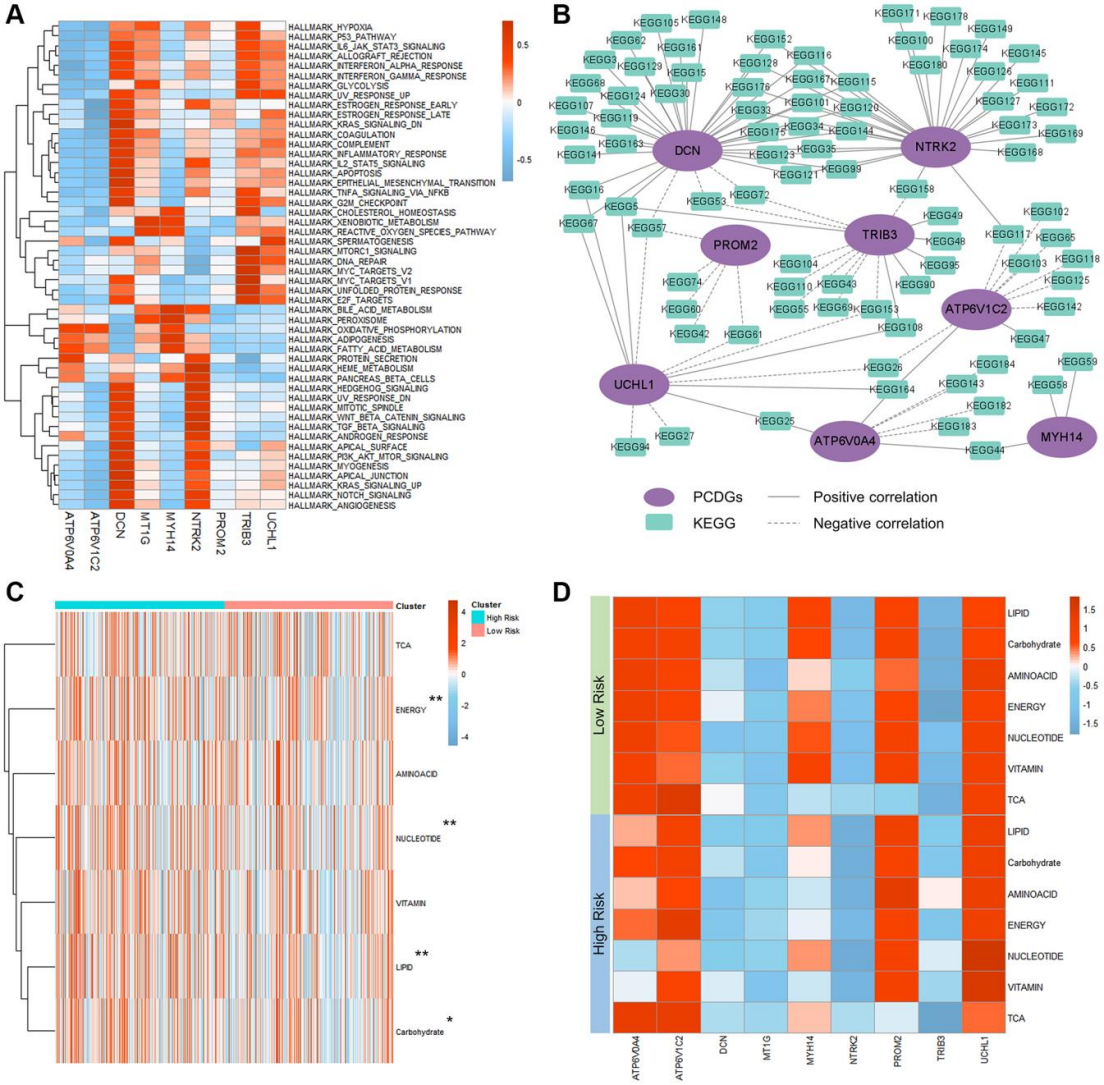
**Mechanisms of PRPCDGs risk signature in KIRC.** (**A**) Heatmap of correlations between PRPCDGs risk signature expression and their activity scores of cancer-associated hallmark pathways. (**B**) Network diagram of the correlations between PRPCDGs’ expression and its highly correlated KEGG pathways. (**C**) Heatmap of activities of 7 metabolic pathways in high- or low-PRPCDGs-risk group from TCGA-KIRC database. (**D**) Heatmap of correlations between the PRPCDGs risk signature and 7 metabolic pathways in two different risk groups from KIRC database.

To explore the cross-talk among these 9 PRPCDGs in KIRC, the expression of them was analyzed by the Pearson correlation coefficient (Supplementary Figure 4A). The results revealed that ATP6V0A4 positively correlated with ATP6V1C2, MYH14 and PROM2, and possessed a negative relation on TRIB3. ATP6V1C2 positively correlated with NTRK2. UCHL1 had a positive correlation with DCN and PROM2. To reveal the potential mechanisms of PRPCDGs in KIRC, the activity scores on KEGG pathways, various cancer-related hallmark pathways and GO terms (molecular functions, biological processes and cell components) and the PCCs of 9 PRPCDGs were computed. The correlations between 9 PRPCDGs and activity scores of cancer-related hallmark pathways are presented in Figure 7A, indicating that different genes responded to different pathways. The correlations with GO terms and KEGG pathways are depicted (Figure 7B and Supplementary Figure 4B–4D, detailed in Supplementary Tables 2–5). The results showed that DCN, NTRK2, TRIB3, ATP6V1C2, ATP6V0A4 and PROM2 had a strong correlation with multiple KEGG pathways, and DCN, UCHL1 and TRIB3 were highly correlated with various GO terms. Interestingly, the expression of DCN performed a strong correlation not with various KEGG pathways but with different GO terms, which meant that DCN possibly played a vital role in KIRC development. Moreover, in the high-PRPCDGs-risk group, metabolism-related pathways were highly enriched and the pathways of energy, nucleotide, lipid and carbohydrate had significantly higher activated levels (Figure 7C, 7D).

### The protumor role of TRIB3 on the development of renal cancer

**Figure 8:**
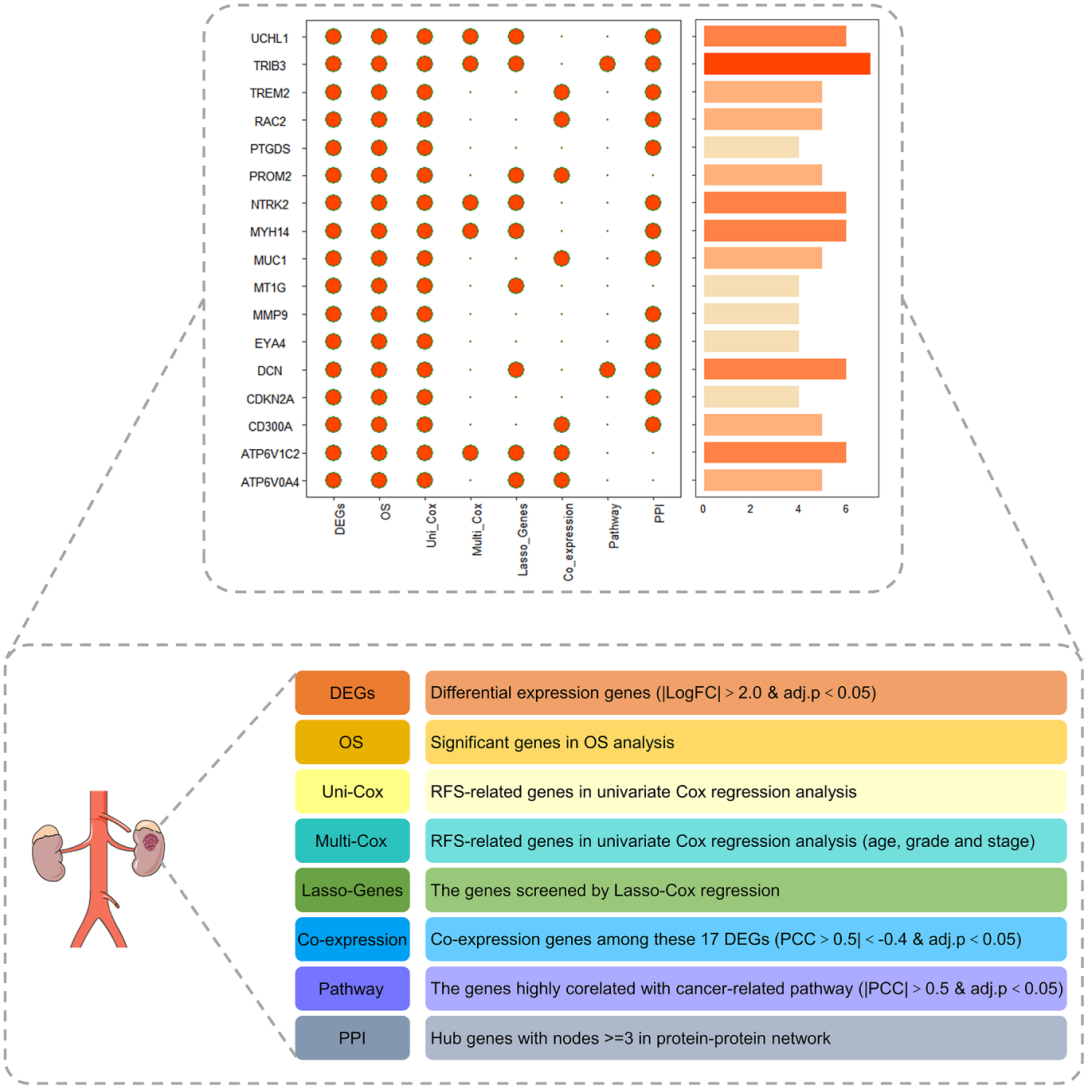
The landscape map of the PRPCDGs risk signature judged by eight indicators, including DEGs, OS, uni-Cox, multi-Cox, Lasso-genes, Coexpression, Pathway and PPI.

To validate the vital role of PRPCDGs in KIRC, eight indicators (DEGs, OS, uni-Cox, multi-Cox, Lasso-genes, Coexpression, Pathway and PPI) were chosen to construct the landscape map of these 17 PRPCDGs, and the scores of these genes were calculated (Figure 8). According to the landscape map, we found that the TRIB3 got the highest score. Therefore, the vital role of TRIB3 in KIRC was further investigated in our study. TRIB3 (Tribbles Pseudokinase 3) is a protein-coding gene in humans. The finding that TRIB3 is highly expressed in RCC has been confirmed in several studies, and the elevated TRIB3 expression in RCC patients is correlated with clinicopathologic features (e.g., tumor grade and stage) of the tumor and prognosis [19].

**Figure 9:**
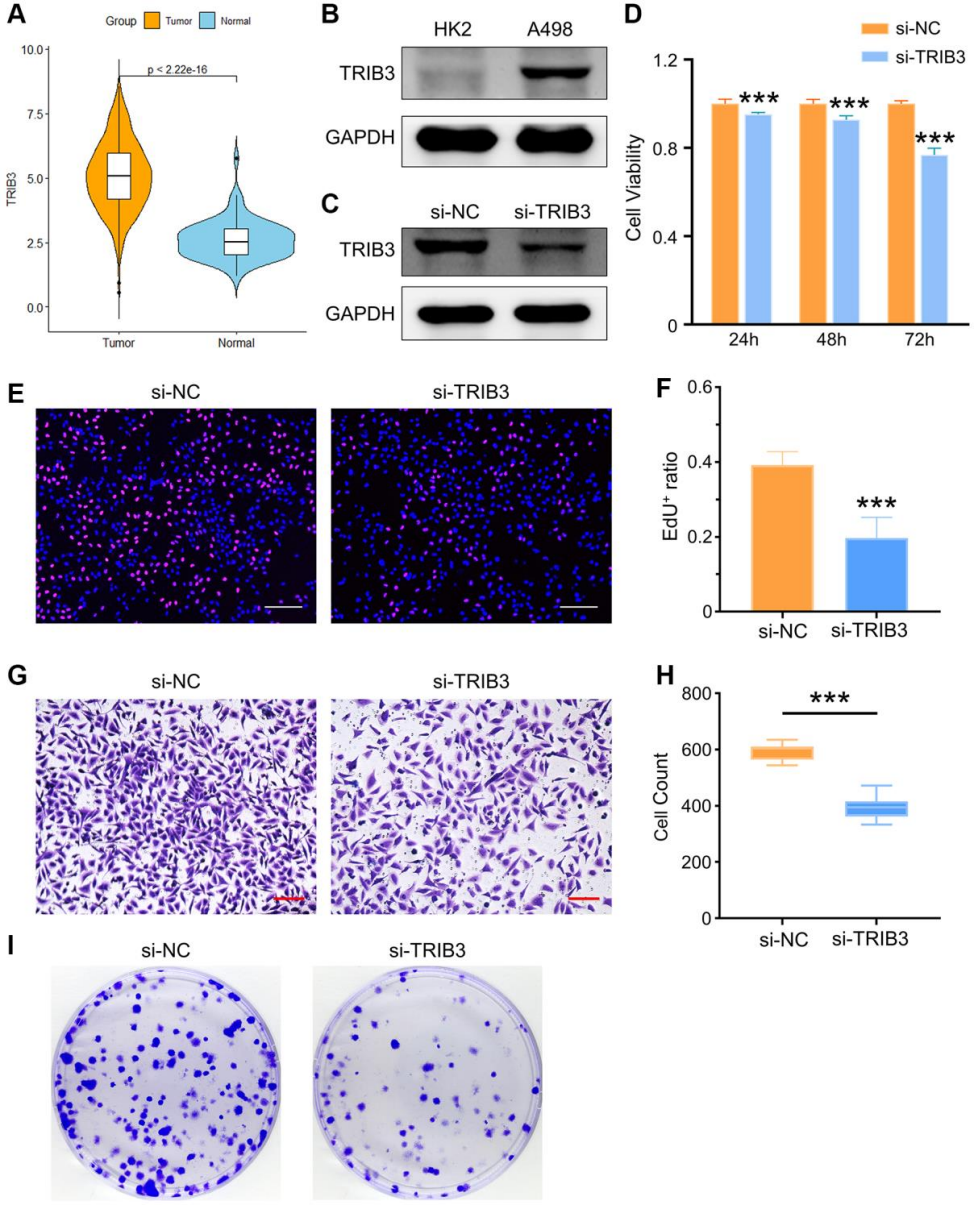
**The role of TRIB3 in tumor development in renal cancer.** (**A**) TRIB3 expression in TCGA-KIRC database. (**B**) TRIB3 expression in HK2 and A498 cells. (**C**) The efficacy of TRIB3 knockdown in A498 cells. (**D**) Cell viability of si-NC- or si-TRIB3-transfected A498 cells. (**E**) Proliferation ability of si-NC- or si-TRIB3-transfected A498 cells tested by EdU staining (scale bar = 200 μm), and the quantified data are shown in (**F**). (**G**, **H**) Invasion capacity of si-NC- or si-TRIB3-transfected A498 cells tested by Transwell assay (scale bar = 200 μm) and the quantified data. (**I**) Cloning assay of A498 cells with si-NC or si-TRIB3 transfection.

**Figure 10:**
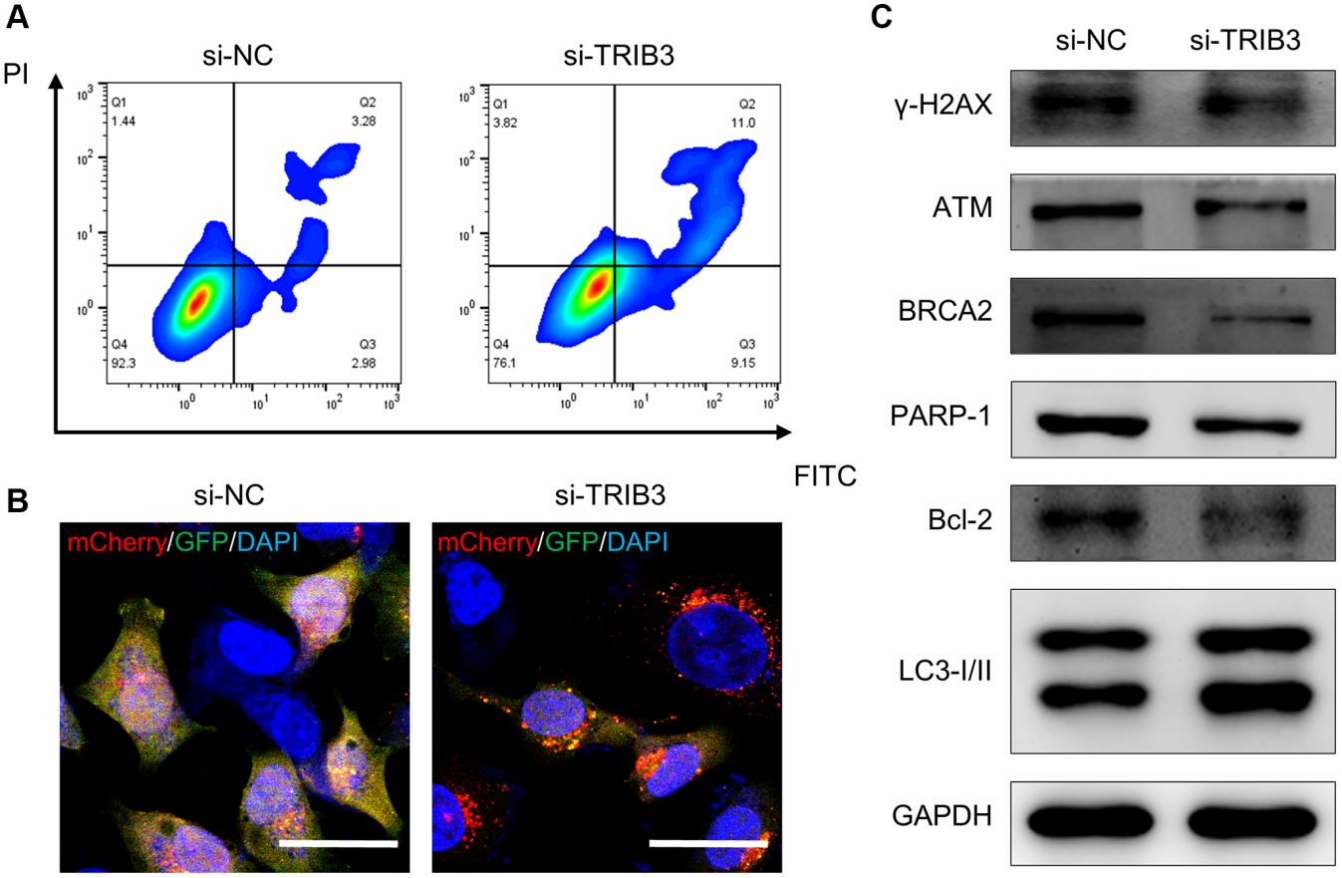
**The role of TRIB3 in tumor development in renal cancer.** (**A**, **B**) Apoptosis and autophagy level of A498 cells with si-NC or si-TRIB3 transfection. (**C**) Protein expression of γ-H2AX, ATM, BRCA2, PARP-1, Bcl-2 and LC3I/II in A498 cells with si-NC or si-TRIB3 transfection.

Here, we found that tumor tissues had a significantly higher TRIB3 expression level than normal tissues (Figure 9A). To validate the differential expression of TRIB3, we used HK-2 (an immortalized proximal tubule epithelial cell line from a normal adult human kidney) and A498 (a cell line with epithelial morphology that was isolated from a kidney cancer patient) cells for exploration. Similarly, elevated expression of TRIB3 was observed in A498 cell line as compared to the expression in HK-2 cell line (Figure 9B). Si-NC- or si-TRIB3-transfected A498 cells were selected to further explore the role of TRIB3 on KIRC. The knockdown of TRIB3 in A498 cells was verified by western blot (Figure 9C). Subsequently, we comprehensively explored the influence of TRIB3 on tumor growth and development by conducting cell viability assays, assessment of cell proliferation and migration capacity and colony formation experiments. Compared with the si-NC group, A498 cells with lower TRIB3 expression exhibited much poorer cell viability at different time points (24 h, 48 h and 72 h post transfection) (Figure 9D), and the proliferation ability, colony formation potential and migration capacity were weakened in the si-TRIB3 group (Figure 9E–9I), which was consistent with the finding that TRIB3 positively affects cell proliferation and invasion in RCC cells, implying that TRIB3 is a tumor-promoting factor in KIRC. To our knowledge, TRIB3 is a key gene in both apoptosis and cellular autophagy processes (Supplementary Table 1). The apoptosis rate of A498 cells was increased from ~6.26% to ~20.15% after transfected with siRNA against TRIB3 (Figure 10A) and bcl-2 expression in A498 cells was downregulated with the knockdown of TRIB3 (Figure 10C), which meant that TRIB3 inhibited the apoptosis process in KIRC. Meanwhile, the elevated autophagic level was characterized by an increased number of red puncta and a decreased number of green puncta in A498 mCherry-GFP-LC3 cells (Figure 10B) and the ratio of LC3II/I was increased in the si-TRIB3 group (Figure 10C). Furthermore, TRIB3 is highly positively correlated with DNA repair (Figure 7A) and severe DNA damage can initiate the apoptotic pathway and ultimately induce cell death. Therefore, the expression of DNA repair-related proteins (γ-H2AX, ATM, BRCA2 and PARP-1) was further examined, showing that defective expression of TRIB3 downregulated the expression levels of these proteins (Figure 10C).

## DISCUSSION

RCC, particularly KIRC, is the most prevalent type of kidney cancer, causing significant global health concerns and mortality. RCC exhibits poor outcomes and an increasing incidence, with approximately 30% of patients developing metastases and the disruptions in metabolism and the immune microenvironment play crucial roles in renal cancer progression. Current treatment approaches such as targeted therapy and immunotherapy have limited efficacy, highlighting the need for new therapeutic targets to effectively manage KIRC.

PCD affects various physiological and pathological processes and its dysfunction plays crucial roles in various cancers [20]. Although apoptosis has been recognized as a key biological process for tumor suppression and several drugs targeting apoptosis have gained some clinical and research momentum, other modes of PCD are as important as apoptosis for the study of potential targets for tumor therapy because of its distinct molecular mechanisms in tumor progression [21]. For example, autophagy, which is responsible for removing damaged or excess cellular components and maintaining intracellular environment homeostasis, is regarded as a double-edged sword in various cancers with both promoting and inhibiting roles in tumors [22]. Similar to autophagy, inflammatory PCD modalities such as necroptosis and pyroptosis have a complicated relationship with cancers, causing both tumor progression and suppression [14, 15]. Ferroptosis, a novel mode of PCD, is involved in a variety of pathological conditions and cancer therapies and is able to kill tumor cells directly, exerting a potential anti-tumor effect [16]. Numerous studies have recovered that higher expression of cuproptosis-associated genes has a positive correlation with poor prognosis in various cancers, but comprehensive mechanistic investigations and multi-omics level analysis are needed to verify this possible relationship [17]. Additionally, with the increasing study of PCD, different types of PCD such as parthanatos [23], entotic cell death [24], netotic cell death [25], lysosome-dependent cell death [26], alkaliptosis [27], oxciptosis [28], anoikis [29], etc., have been discovered and investigated, playing vital roles in tumor development with their distinct characteristics and regulatory mechanisms. Thus, PCD is a vital process in tumorigenesis and progression and provides more potential targets for tumor therapy.

In this study, 1257 genes associated with thirteen PCD modalities (apoptosis, autophagy, pyroptosis, ferroptosis, necroptosis, cuproptosis, parthanatos, entotic cell death, netotic cell death, lysosome-dependent cell death, alkaliptosis, oxciptosis and anoikis) were obtained from previous studies [18] and GSEA database. Forty-one DEGs were selected and primarily enriched in apoptosis, autophagy and lysosome signaling pathways. Subsequently, 17 PRPCDGs that significantly affected patients’ prognosis were screened. Later, the PRPCDGs risk signature, consisting of ATP6V0A4, ATP6V1C2, DCN, MT1G, MYH14, NTRK2, PROM2, TRIB3 and UCHL1, was identified by the LASSO regression analysis according to PRPCDGs’ expression in TCGA-KIRC dataset. From the Pearson correlation coefficient, ATP6V0A4 positively correlated with ATP6V1C2, MYH14 and PROM2, and possessed a negative relation on TRIB3. ATP6V1C2 positively correlated with NTRK2. UCHL1 had a positive correlation with DCN and PROM2. What’s more, mRNA expression of MYH14, ATP6V0A4 and NTRK2 negatively affected the risk scores while the other 6 PRPCDGs showed positive correlations with the scores. Patients with the lower PRPCDGs risk scores in TCGA-KIRC cohort or in the majority of its clinical feature-based subgroups (age, sex, clinical stage and pathological grade) showed more favorable outcomes than those with higher scores. The robust predictive efficacy of this predictive model was also applicable in the E-MTAB-1980 and KIRP datasets. These findings implied that PRPCDGs risk signature possessed a robust predictive efficacy in patients’ prognosis. Moreover, the PRPCDGs risk signature was also illustrated as an independent prognostic factor via analyses of uni- and multi-Cox regression and a nomogram model constructed by multi-Cox regression analysis showed ideal predictive efficacy for the 5-/7-year prognosis of patients. For immunotherapy response, the PD patients presented higher risk scores than CR patients in KIRC and KIRP cohorts, and patients who displayed high PRPCDGs risk scores showed increased stromal and immune scores in tumors. The assessment of immune checkpoint expression revealed that the high-PRPCDGs-risk group presented higher expression levels of CTLA4, PDCD1, TIGIT, CD27, and LAG3 while CD274 was less expressed. A previous study illustrated that CTLA4 blocked with ipilimumab (a blocking antibody of CTLA4) induces cancer regression in some metastatic RCC patients [30]. PDCD1 and CD274 differentially modulate the prognosis in lung adenocarcinoma or squamous cell carcinoma [31], and rational combinations of first series (CTLA-4, PD-1 and PD-L1) and second series (TIGIT, TIM-3 and LAG-3) of inhibitory receptors might be an optimal immunotherapy approach because of the coexpression or/and compensatory mechanisms in various cancers [32]. Therefore, exploring the specific functions and mechanisms of PRPCDGs in KIRC could provide a reference for targeted therapies. In addition, the infiltration and activity of various immune cells were assessed. Treg cells, B cells and macrophages were highly activated in KIRC. Treg cells affect the tumor immune microenvironment by modulating various immune cells such as macrophages, dendritic cells, T cells, NK cells and B cells [33]. High infiltration of Treg cells in RCC tumors is related to unfavorable clinical outcomes [34]. The depletion of B cells therapeutically enhanced the responses of antitumor immunity in certain tumors by reducing the IL-10 secretion of B cells [35]. IL-10 has the ability to induce M2 polarization of tumor-associated macrophages, thus promoting tumor growth in RCC [36]. The activity of Th2 cells is higher in the high-PRPCDGs-risk group. Polarization to Th2 phenotype of CD4^+^ T cells was evident in breast cancer patients in advanced stages and associated with the immunotherapy, prognosis and metastasis in luminal breast cancer [37]. Moreover, the energy, nucleotide, lipid and carbohydrate metabolisms were significantly activated in high-PRPCDGs-risk group. Metabolic reprogramming has gained much attention in the last decade [38]. Elevated exogenous lipid uptake or endogenous synthesis is vital for the survival and proliferation of neoplastic cells. Disturbance in lipid metabolism is a prominent change in RCC and is likely responsible for RCC aggressiveness [39]. Energy metabolism (glycolysis pathway) was reported to be a potential mechanism of RCC progression in bioinformatics analysis [40], and growing evidence supports that targeted nucleotide metabolism can increase anti-tumor immune response through activation of the host immune system [41]. These results suggested that PCD could affect patient prognosis via immunity and metabolism modulation, which also implied that investigations on PCD’s effects on tumor immune microenvironment and metabolism may provide further insights into specific mechanisms of tumor progression and potential molecular biology references for targeted tumor therapies.

Furthermore, to further validate the role of PCD in RCC, a landscape map covering eight indicators (DEGs, OS, uni-Cox, multi-Cox, Lasso-genes, Coexpression, Pathway and PPI) of 17 PRPCDGs was constructed. Among these, TRIB3 received the highest score and was selected as the hub gene. TRIB3 is a protein-coding gene in humans. Several studies have observed that TRIB3 is highly expressed in RCC, and the elevated TRIB3 expression in RCC patients was correlated with clinicopathologic features of the tumor and prognosis [19]. What’s more, TRIB3 could activate the MAPK pathway, leading to an enhancement of cell survival, proliferation and invasion of RCC cells [19]. In our study, TRIB3 was mainly enriched in the cancer-related hallmarks of myc targets V2, mTOR1 signaling, glycolysis, hypoxia, DNA repair and unfolded protein response. TRIB3 was significantly upregulated in tumor tissues in TCGA-KIRC database and its expression in A498 cells was dramatically elevated compared with that in HK2 cells. The knockdown of TRIB3 decreased the proliferation potential, invasion capacity and colony formation ability of A498 cells and enhanced the apoptosis and autophagy process of A498 cells, which indicated that TRIB3 acted as a pro-tumor factor in RCC. Additionally, TRIB3 knockdown led to a lower expression of DNA repair-related proteins, including γ-H2AX, PARP-1, BRCA2 and ATM. TRIB3 has been shown to respond to DNA repair and interact with a variety of DNA repair genes, including BRCA1 and ATM to regulate genome integrity [42]. These proteins are responsible for various DNA damage repair pathways and synergistically maintain genome stability and cell survival. γ-H2AX (the phosphorylated form of H2AX) responds by recruiting DNA damage response proteins to damaged chromatin, and the loss of γ-H2AX leads to DNA double-strand-break repair defects and genome instability [43]. ATM (ataxia telangiectasia-mutated) is mainly involved in the DNA double-strand break repair process [44]. In addition to double-strand break repair, BRCA2 (breast cancer 2) is also closely related to homologous recombination repair [45], while PARP-1 (poly (ADP-ribose) polymerase 1) plays important regulatory roles in single-strand break repair and basic cleavage repair [45]. The inhibition of ATM (inhibited by KU-60019) induced a strong suppressive effect on cell proliferation, migration and ROS-dependent apoptosis in RCC cells [46], and the deactivated BRCA2 could induce apoptosis through TNFα signaling pathway in multiple breast- and leukemic cell lines [47]. The knockdown of PARP-1 dramatically boosted TRA-8-induced apoptosis in pancreatic cancer cells *in vitro* [48]. These results suggested that knockdown of TRIB3 enhanced apoptosis in RCC possibly through the inhibition of the DNA damage repair process.

Together, our study indicated that PCD might affect the prognosis of RCC patients. More investigations on PRPCDGs (such as TRIB3) could get further insights into mechanisms of tumor progression and provide potential targets for RCC treatment.

## METHODS

### Data acquisition

PCD-related genes were obtained from high-quality articles [18]. The gene information is summarized in Supplementary Table 1. The profiles of mRNA expression and clinical data of KIRC and KIRP were acquired from the TCGA (https://xena.ucsc.edu/). E-MTAB-1980, an external RCC data containing clinical data and normalized mRNA expression, was obtained from the Array Express database (https://www.ebi.ac.uk/arrayexpress). The mRNA expression of KIRC and KIRP was normalized and transformed into the form of Log2 (TPM+1). The E-MTAB-1980 was applied for further external analyses.

### Differential expression and enrichment analyses

The limma R package was obtained for screening out the differentially expression genes (DEGs). The Benjamini Hochberg method was utilized for the adjustment of *P*-value. |log2(Fold change)| >2 and adjusted *P*-value < 0.05 were set as the filtering threshold of DEGs. The clusterprofiler R-package was applied for functional and mechanism enrichment analyses of the DEGs based on the Gene Ontology (GO) and Kyoto Encyclopedia of Genes and Genomes (KEGG). GO includes cell components (CC), biological processes (BP), and molecular functions (MF).

### Uni-Cox regression and survival analyses

Based on the survival R package, uni-Cox regression analysis was performed by matching the DEGs’ mRNA expression data with their corresponding clinical data and the efficacy of prediction was evaluated by hazard regression model to determine the PCD genes that significantly affected patient prognosis, also named as PRPCDGs. Using surv_cutpoint R-function from survminer R-package, optimal cut-off value was finally determined to separate single gene expression data, and the Kaplan-Meier survival curves were then depicted for the presentation of the difference in survival rate. The significance was determined via log-rank tests.

### Construction of the PRPCDGs risk signature

Utilizing the createDataPartiton R-function, we separated the TCGA-KIRC dataset into training and testing cohorts at a ratio of 1:1. To construct the prognosis-related risk signature, the LASSO-penalized Cox (LASSO Cox) regression analysis was then conducted based on the glmnet R-package and 10-fold cross-validation in KIRC-training dataset. A 9-gene signature was finally determined. Gene expression and coefficients derived from LASSO Cox regression were used for the establishment of the formula for risk score computation.

PRPCDGs risk score = ATP6V1C2 × 0.41151193 − ATP6V0A4 × 0.21532181 + DCN × 0.11250123 + MT1G × 0.03749527 − MYH14 × 0.10682736 − NTRK2 × 0.09850798 + PROM2 × 0.05556780 + TRIB3 × 0.26818505 + UCHL1 × 0.00498139.

Receiver operating characteristic (ROC) curve analysis was then utilized based on the survivalROC R-package to estimate the risk signature’s predictive accuracy on patients’ prognosis. The median value of risk scores departed the KIRC-training dataset into high- and low-PRPCDGs-risk groups. Kaplan-Meier survival curve was depicted for the determination of survival rate difference between these two risk groups. The same analyses were performed in KIRC-testing dataset and two external cohorts (KIRP and E-MTAB-1980 datasets) for further validation.

### Construction of nomogram

To make the risk score further adjusted by clinical variables covering age, gender, stage, and grade, the variables with statistical significance derived from uni-Cox regression analysis were utilized for the conduct of multi-Cox regression analysis to screen out individual risk variables. We further carry out a stepwise regression method to determine variables. Not until the Akaike information criterion reached the minimum did the analysis stop and the variables were determined. Then a nomogram was constructed utilizing rms R-package to assess the 3-/5-/7- year OS probability. Time-dependent ROC and calibration plots estimated the prediction accuracy and clinical benefits of the nomogram were determined by the decision curve analysis (DCA).

### Coexpression, gene set enrichment and gene set variation analysis

The Pearson correlation coefficient (PCC) among 9 genes was calculated by the rcorr R-function based on Hmisc R-package. The gene sets of hallmark-related cancer pathways, GO (including molecular functions, cell components, biological process), and KEGG pathways were obtained from molecular signatures database (MSigDB) (https://www.gsea-msigdb.org/gsea/msigdb/index.jsp). Then mRNA expression data and the gene sets were utilized for calculation of activity scores among various terms based on the Gene Set Variation Analysis (GSVA) R package. Then, we computed the PCC between PCPGs’ expression and activity score of every term. We think that |PCC| >0.3 and *P*-value < 0.05 represent the existing correlation between the two groups of data.

### Immune infiltration analysis and metabolic reprogramming

Twenty-four gene sets on immune cells were obtained from the previous literature [49] and the activity scores were calculated by the method of single-sample gene set enrichment (ssGSEA) from GSVA R-package. GSVA is an unsupervised and non-parametric method for estimating gene set enrichment variation through the expression dataset of the samples [50]. We utilized the estimate R-package to assess the immune and stromal scores. Cell Type Identification by Estimating Relative Subsets of RNA Transcripts (CIBERSORT) was then adopted for assessment of immune infiltration among 22 types of immune cells. Metabolic signatures were also obtained from the previous research and the same ssGSEA analysis was conducted.

### Cell culture

HK-2 and A498 cells were cultured in Dulbecco's Modified Eagle Medium (DMEM) (Gibco, USA) supplemented with 10% fetal bovine serum (Gibco, USA) and 1% penicillin/streptomycin liquid (Solarbio, China).

### Knockdown of TRIB3

SiRNA specific to TRIB3 (si-TRIB3) and siRNA for negative control (si-NC) were purchased from GenePharma (China). When the confluence reached ~30%, with the application of Lipofectamine™ 3000 kit (Invitrogen, USA), A498 cells were transfected with si-TRIB3 or si-NC. The medium was changed 24 h after transfection. The sequences of si-TRIB3 were as follows: TRIB3: 5′-GGAAGAAGCGGUUGGAGUUTT-3′ (sense), 5′-AACUCCAACCGCUUCUUCCTT-3′ (antisense).

### Cell viability assessment

After the transfection of si-TRIB3 and si-NC, the Cell Count Kit-8 assay was conducted at 24, 48 and 72 h to assess the cell viability of A498 cells. The O.D. values were normalized to the si-NC group.

### Proliferation, cloning and invasion assays

After the transfection of si-TRIB3 and si-NC, A498 cells were plated into the confocal dishes, 6-cm cell culture dishes and chambers of cell culture inserts. Click-iT EdU cell proliferation assay (Thermo Fisher Scientific, USA) was used for cell proliferation test and a laser confocal microscope was used for the observation of cells. For the clone and invasion assay, cells were stained with crystal violet ammonium oxalate solution (Solarbio, China). The culture dishes were imaged and the chambers were observed via microscopy.

### Apoptosis assay

After the transfection of si-TRIB3 and si-NC, A498 cells were harvested with 0.25% trypsin digestion solutions (without phenol red) (Solarbio, China) and were then stained with an Annexin V-FITC Apoptosis Detection Kit (Solarbio, China). The fluorescent signal of cells was collected by a flow cytometer.

### Autophagy detection

The effect of TRIB3 on autophagic flux was detected in A498 mCherry-GFP-LC3 cells. Briefly, after the transfection of si-TRIB3 and si-NC, A498 mCherry-GFP-LC3 cells were plated into the confocal dishes for 24 h. Then the cells were fixed by 4% paraformaldehyde (PFA) and incubated with DAPI (Solarbio, China). Finally, the tandem fluorescent LC3 puncta was captured with a laser confocal microscope.

### Western blot

After the harvest of total proteins in A498 cells, equal amounts of proteins (30 μg) were used for SDS-PAGE electrophoresis. Then, the proteins were transferred onto a PVDF membrane where they were totally blocked with a blocking buffer. After the incubation with primary antibodies (anti-GAPDH, anti-TRIB3, anti-γ-H2AX, anti-ATM, anti-BRCA2, anti-PARP-1, anti-Bcl-2 and anti-LC3I/II) (Proteintech, China) at 4°C overnight, the membranes were incubated with the secondary antibodies at room temperature for 2 h, Bio-Rad ChemiDoc XRS chemiluminescence system (Bio-Rad Inc., USA) was utilized to detect the western blots.

### Statistical analysis

The unpaired *t*-test was utilized to determine the mean value differences between two groups of data. Relations between various variables and patients’ prognosis were assessed via the analyses of uni- and multi-Cox regression and correlation coefficients between the two groups were computed with the application of Pearson method. *P*-value < 0.05 was considered significant in all analyses. ^*^P-value < 0.05, ^**^P-value < 0.01, ^***^P-value < 0.001, ^****^P-value < 0.0001.

### Data availability statement

All datasets generated for our research are introduced in the article.

## Supplementary Materials

Supplementary Figures

Supplementary Table 1

Supplementary Table 2

Supplementary Table 3

Supplementary Table 4

Supplementary Table 5
