# Supplementary material for: A prognostic risk model for programmed cell death and revealing TRIB3 as a promising apoptosis suppressor in renal cell carcinoma
Source: Aging (Albany NY). 2023 Nov 22;15(22):13213–38. doi: 10.18632/aging.205237 (PMC10713411; doi:10.18632/aging.205237)
Supplement: Supplementary Figures [file aging-15-205237-s001.pdf]

SUPPLEMENTARY FIGURES

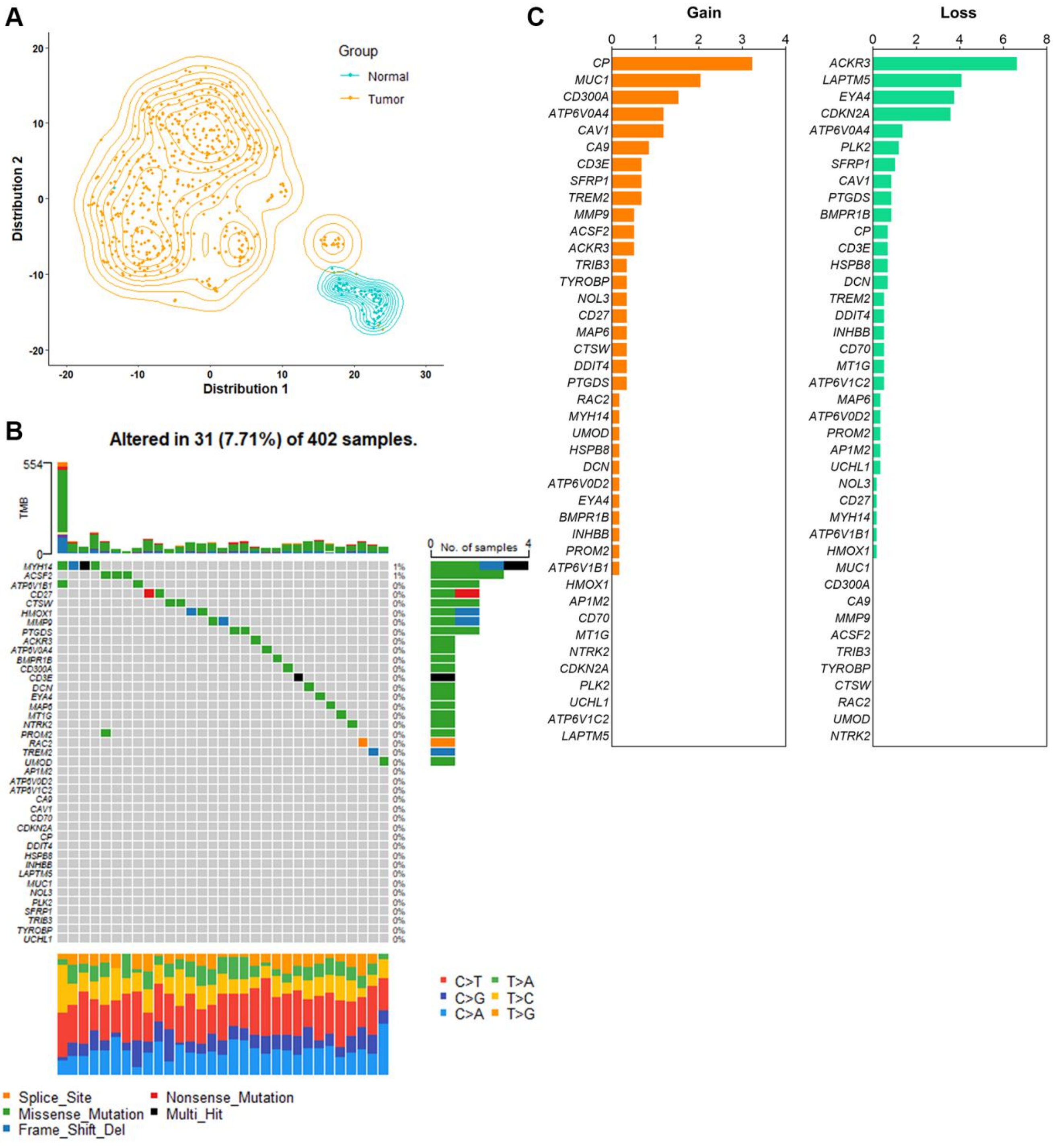

Supplementary Figure 1. (**A**) Distribution of tumor and normal groups calculated by t-SNE analysis in TCGA-KIRC database. Tumor mutation burden (TMB) (**B**) and copy number variations (CNVs) (**C**) analyses of these PCD-related DEGs in KIRC database.

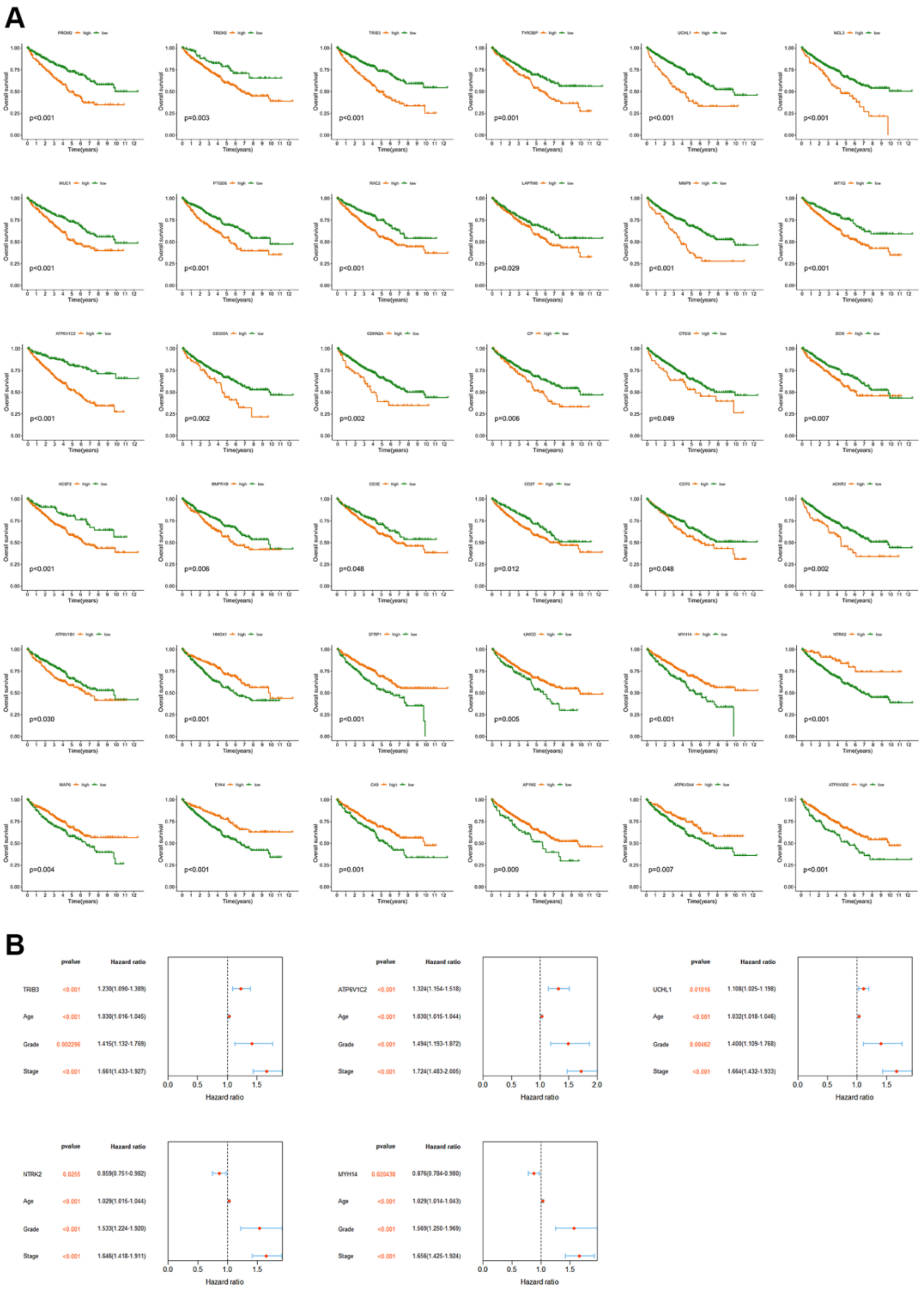

Supplementary Figure 2. (**A**) Kaplan-Meier survival curves of these prognosis-related DEGs based on their expression in TCGA-KIRC database. (**B**) Forest plots of TRIB3, ATP6V1C2, UCHL1, NTRK2 and MYH14 depicted by multi-Cox regression analysis (adjusted by age, grade and stage).

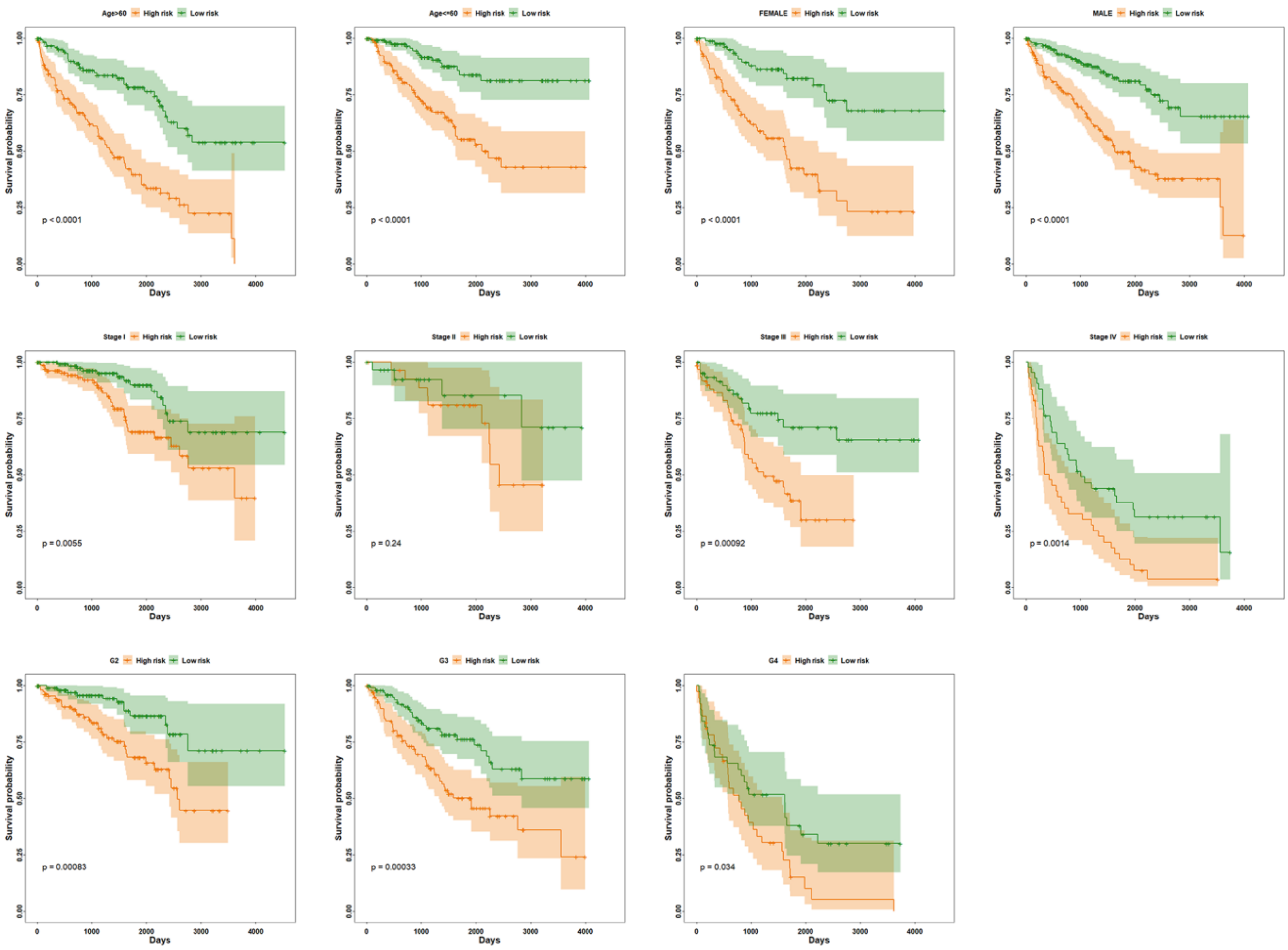

Supplementary Figure 3. Validation of PRPCDGs risk signature's prognostic predictive performance on clinicopathologic features (age, gender, stage and grade) with Kaplan-Meier analysis in TCGA-KIRC cohort.

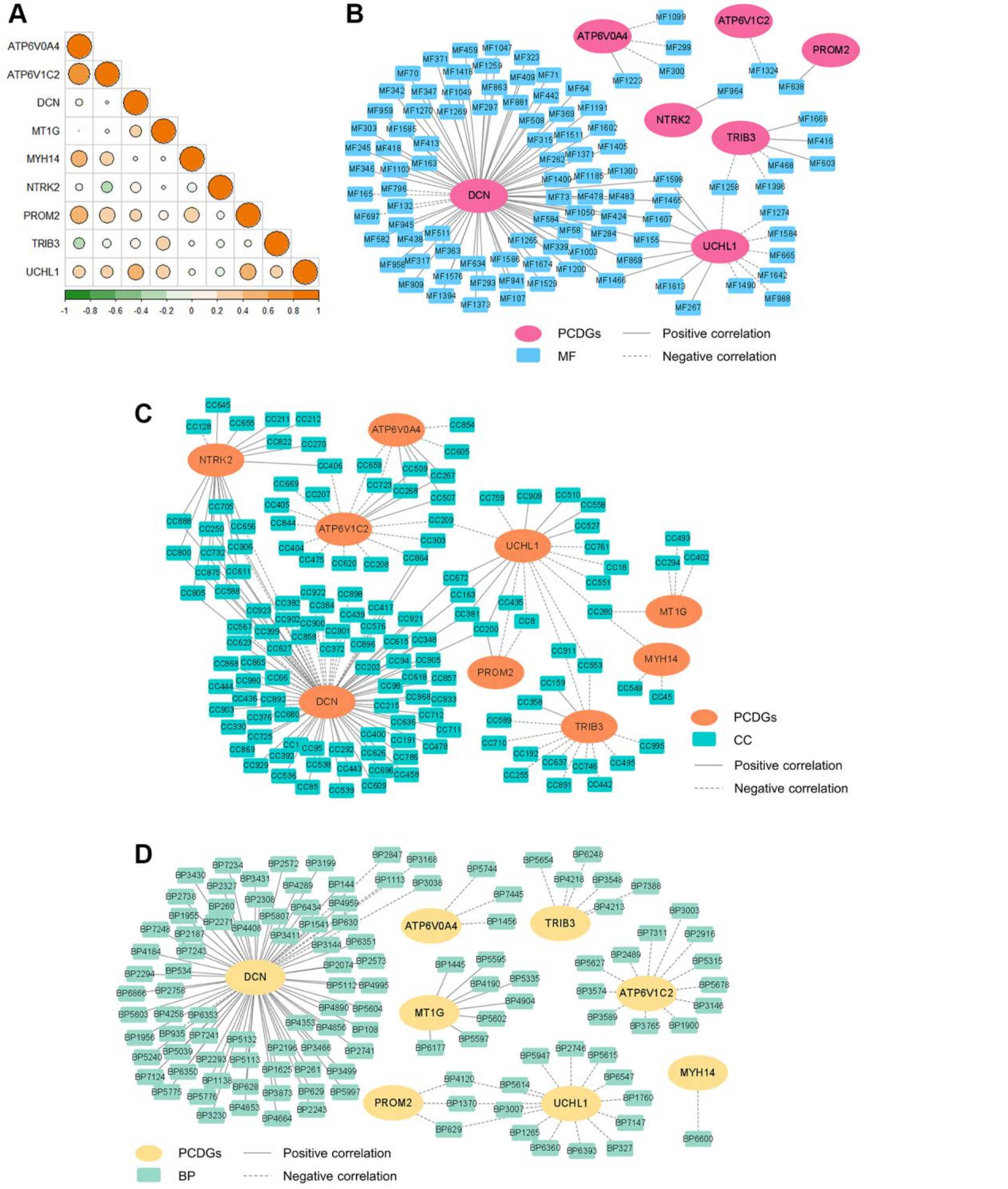

Supplementary Figure 4. (**A**) Correlation diagrams of the correlations among the 9-gene PRPCDGs risk signature expression in TCGA-KIRC database (positive correlations in orange dots, negative correlations in green and the size of the dots shows the significance level). (**B**–**D**) Network diagrams of the correlations between PRPCDGs' expression and its highly correlated GO terms.
